# Estimates of recent and historical effective population size in turbot, seabream, seabass and carp selective breeding programmes

**DOI:** 10.1186/s12711-021-00680-9

**Published:** 2021-11-06

**Authors:** María Saura, Armando Caballero, Enrique Santiago, Almudena Fernández, Elisabeth Morales-González, Jesús Fernández, Santiago Cabaleiro, Adrián Millán, Paulino Martínez, Christos Palaiokostas, Martin Kocour, Muhammad L. Aslam, Ross D. Houston, Martin Prchal, Luca Bargelloni, Kostas Tzokas, Pierrick Haffray, Jean-Sebastien Bruant, Beatriz Villanueva

**Affiliations:** 1 Departamento de Mejora Genética Animal, INIA-CSIC, Ctra. de La Coruña, km 7.5, 28040 Madrid, Spain; 2 Centro de Investigación Mariña, Facultade de Bioloxía, Universidade de Vigo, 36310 Vigo, Spain; 3 Departamento de Biología Funcional, Universidad de Oviedo, C/ Julián Clavería s/n, 33006 Oviedo, Spain; 4 CETGA, Cluster de Acuicultura de Galicia, Punta do Couso s/n, 15695 Aguiño-Ribeira, Spain; 5 Geneaqua, 27002 Lugo, Spain; 6 Departament of Zoology, Genetics and Physical Anthropology, Universidade de Santiago de Compostela, 27002 Lugo, Spain; 7 The Roslin Institute and Royal (Dick) School of Veterinary Studies, University of Edinburgh, Easter Bush, Midlothian, EH25 9RG UK; 8 South Bohemian Research Center of Aquaculture and Biodiversity of Hydrocenoses, Faculty of Fisheries and Protection of Waters, University of South Bohemia in České Budějovice, Zátiší 728/II, 389 25 Vodňany, Czech Republic; 9 Nofima AS, P.O. Box 210, 1431 Ås, Norway; 10 Universitá degli Studi di Padova, Via 8 Febbraio 1848, 2, 35122 Padova, PD Italy; 11 Andromeda Group SA, Leof. Lavriou 99, 190 02 Peania, Greece; 12 SYSAAF, Station LPGP/INRAE, Campus de Beaulieu, 35042 Rennes, France; 13 Ferme Marine De Douhet, Route du Douhet, 17840 La Brée-les-Bains, France

## Abstract

**Background:**

The high fecundity of fish species allows intense selection to be practised and therefore leads to fast genetic gains. Based on this, numerous selective breeding programmes have been started in Europe in the last decades, but in general, little is known about how the base populations of breeders have been built. Such knowledge is important because base populations can be created from very few individuals, which can lead to small effective population sizes and associated reductions in genetic variability. In this study, we used genomic information that was recently made available for turbot (*Scophthalmus maximus*), gilthead seabream (*Sparus aurata*), European seabass (*Dicentrarchus labrax*) and common carp (*Cyprinus carpio*) to obtain accurate estimates of the effective size for commercial populations.

**Methods:**

Restriction-site associated DNA sequencing data were used to estimate current and historical effective population sizes. We used a novel method that considers the linkage disequilibrium spectrum for the whole range of genetic distances between all pairs of single nucleotide polymorphisms (SNPs), and thus accounts for potential fluctuations in population size over time.

**Results:**

Our results show that the current effective population size for these populations is small (equal to or less than 50 fish), potentially putting the sustainability of the breeding programmes at risk. We have also detected important drops in effective population size about five to nine generations ago, most likely as a result of domestication and the start of selective breeding programmes for these species in Europe.

**Conclusions:**

Our findings highlight the need to broaden the genetic composition of the base populations from which selection programmes start, and suggest that measures designed to increase effective population size within all farmed populations analysed here should be implemented in order to manage genetic variability and ensure the sustainability of the breeding programmes.

**Supplementary Information:**

The online version contains supplementary material available at 10.1186/s12711-021-00680-9.

## Background

The success of any breeding programme depends critically on how the base population of breeders is built, since the genetic variability that is initially available in the founders will affect the genetic progress achieved in the subsequent selection programme [1–3]. This is particularly important in aquaculture because, given the high fecundity of fish species, base populations can be created from very few individuals, which would lead to small effective population sizes (*N*_*e*_) and therefore, to high rates of loss of genetic variability, high rates of inbreeding and restricted long-term selection responses.

With the rapid development of genomic tools, temporal series of *N*_*e*_ can be estimated for generations before pedigree recording began. This is of great importance in aquaculture species to determine the impact of domestication on the genetic variability present in the base populations and the potential long-term response to selection. Genomic estimates of *N*_*e*_ are obtained based on the linkage disequilibrium (LD) approach [4], and different methods have been developed to estimate this parameter across generations. These methods have assumed that the *N*_*e*_ of a particular generation in the past can be estimated from LD between pairs of single nucleotide polymorphisms (SNPs) separated by a specific distance [5]. However, this assumption implies that the demographic events that occurred in that particular generation do not affect subsequent generations, and the method only holds for linear changes in population size [5]. To circumvent this problem, Santiago et al. [6] have recently developed an approach where the LD spectrum for the whole range of recombination rates between all pairs of SNPs is taken into account for estimating *N*_*e*_ in consecutive generations, and this allows the detection of drastic changes in population size.

In spite of the importance of estimating *N*_*e*_, estimates of this parameter are scarce for most aquaculture species. In this study, we used genomic information that was recently produced for important fish species in European aquaculture (turbot, gilthead seabream, European seabass and common carp) to obtain recent and historical estimates of *N*_*e*_ for commercial populations, using the novel method developed by Santiago et al. [6]. These estimates are useful to evaluate the current genetic status of the populations and to identify past changes in *N*_*e*_ potentially associated with domestication or with the establishment of selective breeding programmes.

## Methods

### Data

Data were derived from broodstock (and their offspring) sampled in 2014 from different European breeding programmes for turbot, gilthead seabream, European seabass and common carp within the framework of the FISHBOOST project (www.fishboost.eu) (Table 1). Unrelated broodstock were mated and their offspring were used for different experimental purposes. Genomic information was available for both parents and their offspring. Genotypes were obtained using reduced representation genotyping approaches [specifically RAD sequencing, (RAD-seq)]. The species’ linkage maps and reference genomes were used to map the SNPs [7–10]. Details on the number of samples and SNPs available for each population analysed are summarised in Table 1. Genotyping and filtering details are described elsewhere for turbot [7], seabream [8], seabass [9] and carp [10]. Imputation of missing genotypes, which was only performed for turbot, was carried out using the software BEAGLE 4.1 [11].

**Table 1:** Description of samples and genomic information for the populations analysed

Population	*N*_*off*_	*N*_*par*_ (♂, ♀)	*n*_*snp*_	*n*_*lg*_	cM	Mb	*d*
Turbot	1391	46 (23, 23)	18,097	22	1403	568	32
Seabream_A	724	117 (57, 59)	15,184	24	1406	790^a^	19
Seabream_F	881	107 (71, 22)	21,701	24	1970	790^a^	28
Seabass	1308	65 (48, 17)	8014	24	1373^a^	577	14
Carp	1349	60 (40, 20)	12,311	50	3944	1830^a^	7

Number of offspring (*N*_*off*_) and parents (*N*_*par*_, including sires and dams) with genotypes available, number of SNPs (*n*_*snp*_) and linkage groups (*n*_*lg*_), genetic (cM) and physical (Mb) genome size and resulting SNP density (*d*, in SNPs per Mb) for each population

^a^Estimates taken from the literature ([44] for turbot; [45] for seabream; [46] for seabass; [47] for carp)

Turbot samples were obtained from an experimental population of Atlantic origin maintained at CETGA (Aquaculture Cluster of Galicia, Spain) through hierarchical matings. For gilthead seabream, data came from one of the four genetically linked yearly cohorts of the breeding nuclei of the Andromeda Group SL (Greece) and Ferme Marine de Douhet (FMD, France), where the main breeding objectives in the selection programmes are growth and body shape. The Andromeda programme applies mass spawning, while the FMD programme applies partial factorial mating designs [8]. European seabass samples came also from one of the four linked yearly cohorts of the FMD breeding nucleus, where the breeding objectives are growth and body shape. In this programme, partial factorial matings are also applied [9]. Finally, for common carp, samples were obtained from the Amur Mirror carp (Vodňany line), which was recently created at the University of South Bohemia in České Budějovice. For this line, F_1_ offspring were obtained from crosses between females from a cultured population (originating from Hungary and Germany) with a mirror phenotype for scaliness and males from a wild population (from the Amur river, Siberia) with a scaly phenotype. The Amur Mirror strain was founded from F_2_ crosses by selecting offspring that had the mirror phenotype. The population used in this study was obtained by artificial fertilization that involved four blocks of full factorial crosses each comprising five dams and ten sires [10].

### Estimation of linkage disequilibrium and effective population size

Estimates of LD between pairs of loci and temporal estimates of *N*_*e*_ were obtained using the software GONE and its auxiliary programs developed by Santiago et al. [6] (available in https://github.com/esrud/GONE). Squared correlations between allele frequencies of pairs of SNPs (*r*^2^; [12]) were obtained for all pairs of SNPs within each linkage group (chromosome). Category bins for different ranges of genetic distances (in Morgans) between SNPs were built and the average values of *d*^2^ (the average of *r*^2^ values between pairs of SNPs weighted by their variance in allele frequences; [13]) were obtained for each bin. The method involves a genetic algorithm to infer the historical series of *N*_*e*_ in the population that minimises the sum of the squared differences between the observed values of *d*^2^ of the bins and those predicted considering different demographic histories. The analyses assumed that phase is unknown and the genetic distances between SNPs were corrected by Haldane’s function. For the remaining software options, the default values were used. In order to compare our results with those of other studies, patterns of LD measured as *r*^2^ across physical distance were represented for the populations for which the physical position of SNPs was available on the reference genome assemblies (i.e. turbot GCA_003186165.1 and seabass GCA_000689215).

For the sake of comparison, temporal estimates of ancestral *N*_*e*_ were also obtained using the previous method of Hayes et al. [5] as implemented by Saura et al. [14]. Although both the GONE method and that of Hayes et al. [5] are based on the well known relationship between LD and *N*_*e*_ [4], the main difference between them is that the former assumes constant *N*_*e*_ or linear changes in *N*_*e*_.

## Results

**Fig. 1:**
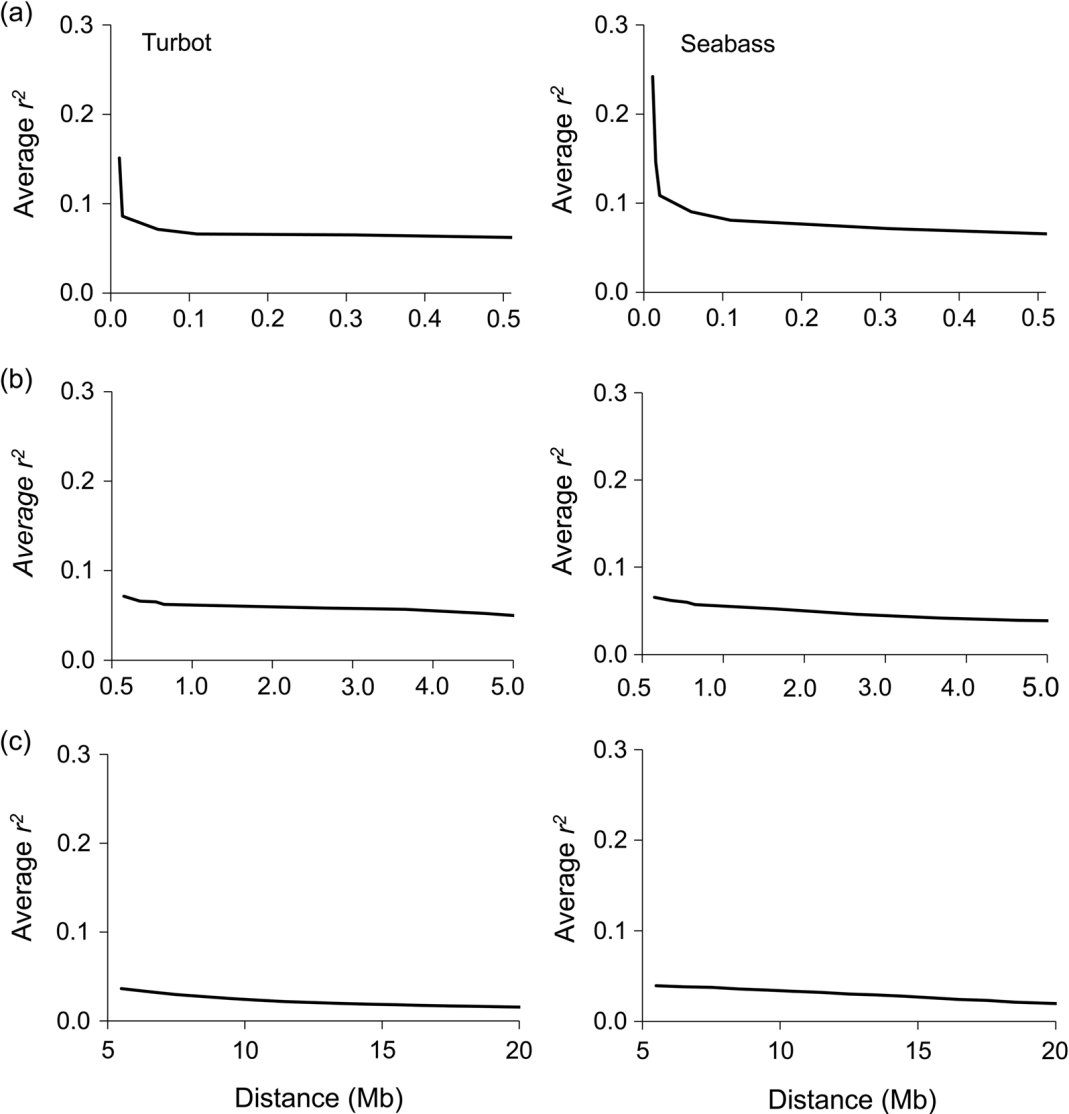
Decay of average linkage disequilibrium across chromosomes measured as *r*^2^ against physical distance. Physical distance in terms of fragment length is indicated in Mb for the species for which a physical map is available; i.e. turbot (left panels) and seabass (right panels). Three different distance categories are represented: **a** from 0.0 to 0.5 Mb; **b** from 0.5 to 5 Mb; **c** from 5 to 20 Mb

The pattern of LD decay with physical distance that was computed with offspring data for turbot and seabass is represented in Fig. 1. Overall, the average LD (*r*^2^) between SNPs separated by short distances (< 0.01 kb) was moderately low (0.15 for turbot and 0.24 for seabass) and decreased rapidly with physical distance. The average *r*^2^ was reduced by half in both cases for distances shorter than 5 kb.

Estimates of recent *N*_*e*_ were equal to or less than 50 fish in all cases. When using offspring data, *N*_*e*_ of 31 for turbot, 46 for seabream_A, 32 for seabream_F, 40 for seabass and 33 for carp were found, and when using parents data, the corresponding values were 26, 50, 30, 32 and 15, respectively.

**Fig. 2:**
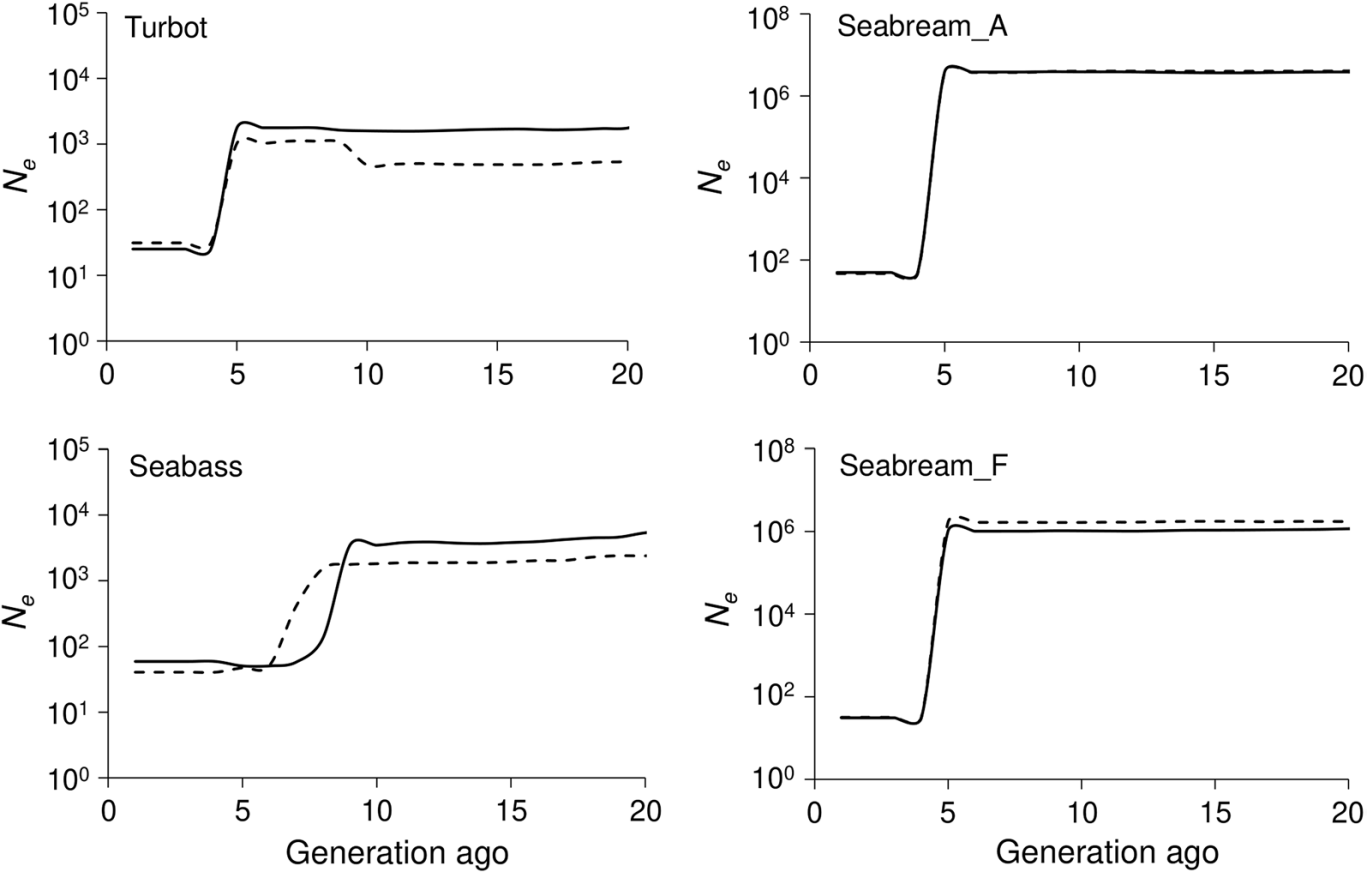
Estimates of *N*_*e*_ (logarithmic scale) across the last 20 generations for each population analysed. Straight lines represent estimates obtained using data from parents and dashed lines represent estimates obtained using data from offspring

Estimates of historical *N*_*e*_ were larger than 1000 fish for about 20 generations ago in all species. However, important drops were observed about five generations ago for turbot and seabream and about eight to nine generations ago for seabass, using data from parents or from offspring (Fig. 2 and see Additional file 1: Fig. S1). The two populations of seabream showed a similar pattern of *N*_*e*_ decay. Estimates of ancestral *N*_*e*_ are not provided for carp since the Amur Mirror strain comes originally from crosses of several strains, and under a scenario of strong and recent population admixture, the method to estimate historical *N*_*e*_ is not conceptually applicable. However, estimates of contemporary *N*_*e*_ can be obtained in this case, although the estimates are likely to be biased downwards because of population admixture [15].

The LD method of Hayes et al. [5] led to linear trends in historical *N*_*e*_, as expected (see Additional file 2: Fig. S2), which contrasts with the drastic drops shown in Fig. 2. Historical values estimated with this method for the earliest generation shown (generation 100) were smaller than 1000 individuals, i.e. much smaller than those obtained by GONE in Fig. 2. However, recent *N*_*e*_ values with the same method (44 for turbot, 33 for seabass, 51 for seabream_A, and 49 for seabass_F) were of the same order of magnitude as those obtained with the method of Santiago et al. [6] and are shown in Fig. 2.

## Discussion

In this study, recent and historical *N*_*e*_ estimates were obtained for farmed populations of important European aquaculture species (turbot, gilthead seabream, European seabass and common carp), using genome-wide SNP data from RAD-seq, and a novel accurate method based on LD measures [6]. Our results revealed that recent *N*_*e*_ for all the analysed populations were small and that important drops in ancestral *N*_*e*_ occurred in these populations about five to nine generations ago.

Recent *N*_*e*_ estimates for all the analysed populations were equal to or less than 50 fish. A value around 50 is considered to fit the minimum value recommended to avoid severe inbreeding depression and retain fitness in the short-term [16–18]. However, our *N*_*e*_ estimates for seabream and seabass could be slightly underestimated given that the data used came from breeding schemes with overlapping generations and the method asumes discrete generations [6, 19].

In general, the magnitude of our recent estimates of *N*_*e*_ was within the range of those found in other farmed fish populations of different species [20–30], although there are exceptions [31]. For instance, the estimate of *N*_*e*_ in the GIFT (Genetically Improved Farmed Tilapia) selection programme in which the creation of the base population was carefully planned, was equal to 88 after seven generations of selection for growth rate [31]. The small estimates of *N*_*e*_ obtained for the farmed populations analysed here contrast with the large estimates (> 1000) found for wild populations of turbot, seabass and seabream [32–34]). Although estimates of *N*_*e*_ for the wild common carp are not available, genetic variability analyses have shown that they are smaller in farmed than in wild strains [35, 36].

Estimates of historical *N*_*e*_ for all the analysed populations revealed important drops occurring about five to nine generations ago. We obtained similar results using data from the reduced number of parental samples or from the more extensive number of offspring samples (Fig. 2). The power of the method to detect fluctuations in *N*_*e*_ is proportional to the product of the sample size and the square root of the number of markers divided by *N*_*e*_, and the minimum value to ensure accurate estimations of *N*_*e*_ is 100 [6]. Using parental samples, the value was much larger than 100, and thus estimates obtained from parents were as reliable as those obtained from offspring.

A drop in *N*_*e*_ and the consequent drop in genetic variability in farmed populations can occur during the establishment of the base population (founder effect) but also in subsequent generations of selection if there is no optimal inbreeding control. Some caution must be taken in the interpretation of the drops observed as they could also be a consequence of population admixture or of the use of inaccurate genetic maps [6]. Nevertheless, our results are highly consistent with the information about the origin of broodstock and how these programmes have been run. Although limited, the available information suggests that the domestication of turbot, gilthead seabream and European seabass started around the 1970s, and that selective breeding programmes for increasing growth rate started in the 1990s [37], with approximately four to six generations of selection to date for the populations analysed here. Under this broad context, our estimates of historical *N*_*e*_ suggest that the combination of both domestication and the start of selection programmes is the most likely explanation for the important recent drops inferred in the populations analysed. Both events may have occurred too close in time to be disentangled by the method.

Our results reflect a moderately low LD between SNPs that are separated by very short distances in turbot and seabass populations. In addition, a very fast LD decay with physical distance was observed in both populations. In fact, *r*^2^ decreased by half at distances shorter than 0.02 Mb and it was maintained when the distance increased by one order of magnitude. At distances longer than 10 Mb, *r*^2^ reached values lower than 0.05. Similar LD values have been reported for coho salmon [29] and Nile tilapia [38] at short distances but the rate of decrease in LD was much slower than those observed here for turbot and seabass. Much higher values of LD (> twofold for short distances) have been reported in farmed populations of Atlantic salmon [28, 39, 40] and rainbow trout [30], with also LD remaining higher over much longer distances. These results may suggest that higher LD values are observed in populations with a longer history of artificial selection.

As already mentioned, an important limitation of the LD method of Hayes et al. [5] to estimate historical *N*_*e*_ is that it only holds for linear changes in population size. Indeed, previous studies applying this method have observed linear trends in *N*_*e*_ over time [28–31], as we observed when reanalysing our data applying this method (see Additional file 2: Fig. S2). As reflected in our results, the method by Santiago et al. [6] provides in this case, a more precise view of the drastic changes in the historical *N*_*e*_, such as those observed in Fig. 2. Another difference between the results of the two methods concerns the large discrepancy between the historical estimates of *N*_*e*_ (see Additional file 1: Fig. S1 and Additional file 2: Fig. S2). In order to shed some light on this issue, we carried out computer simulations under a scenario that mimics the pattern observed in Fig. 2 (see Additional file 3: Fig. S3 for results and simulation details). In the simulations, a large population with a constant size *N* of 1000 or 10,000 suddenly drops to *N* = 100 or 50 individuals in the last ten or five generations, respectively. We repeated this simulation 20 times and carried out analyses with the methods of Santiago et al. [6] (using GONE) and Hayes et al. [5]. The simulations show that the method of Hayes et al. [5] does not reflect the sudden drop in population size, and that it gives very downwardly biased estimates of the historical size. The simulations also show that the ancestral *N*_*e*_ obtained by GONE can be overestimated, particularly when the size of the ancestral population is large. Thus, the large observed values of ancestral *N*_*e*_ shown in Fig. 2 and Additional file 2: Fig S2 should be taken with caution, since they can be overestimations. In any case, GONE is able to detect the drastic change in *N*_*e*_ as reflected in both figures.

## Conclusions

In summary, our results suggest that the current *N*_*e*_ of the commercial populations analysed here are, in general, below the critical value of 50 individuals that is recommended to ensure short-term sustainability of selection programmes. Series of historical *N*_*e*_ reveal important drops probably due to domestication and the start of breeding programmes. Our findings highlight the need for broadening the genetic composition of base populations from which selection programmes start and suggest that measures to increase *N*_*e*_ within all the farmed populations analysed here should be implemented. These measures include increasing the number of parents selected, conducting artificial fertilization and applying single-pair rather than mass spawning [41], and if possible implementing optimal contribution selection [42, 43], to maximise genetic gain while restricting the rate of inbreeding. In cases where these interventions are not sufficient to increase *N*_*e*_ above the critical value, another option could be to interchange genetic material from different genetically improved stocks.

## Supplementary Information

**Additional file 1.** Estimates of *N*_*e*_ (logarithmic scale) across the last 100 generations for each population analysed. Straight lines represent estimates obtained using data from parents and dashed lines represent estimates obtained using data from offspring.

**Additional file 2.** Estimates of *N*_*e*_ (logarithmic scale) obtained with the LD method of Hayes et al. [5] across the last 100 generations for each population analysed.

**Additional file 3.** Estimates of *N*_*e*_ (logarithmic scale) obtained by computer simulations with the LD methods of Hayes et al. [5] and Santiago et al. [6], using the software SLiM3 [48].

## Data Availability

The aligned reads for carp in the format of bam files were deposited in the National Centre for Biotechnology Information (NCBI) repository under project ID PRJNA414021. For seabass, the sequence reads were deposited at the NCBI Sequence Read Archive (SRA) under the accession number PRJNA407892.
