# Supplementary material for: Estimates of recent and historical effective population size in turbot, seabream, seabass and carp selective breeding programmes
Source: Genet Sel Evol. 2021 Nov 6;53:85. doi: 10.1186/s12711-021-00680-9 (PMC8572424; doi:10.1186/s12711-021-00680-9)

Estimates of N_e (logarithmic scale) across the last 100 generations for each population analysed. Straight lines represent estimates obtained using data from parents and dashed lines represent estimates obtained using data from offspring.

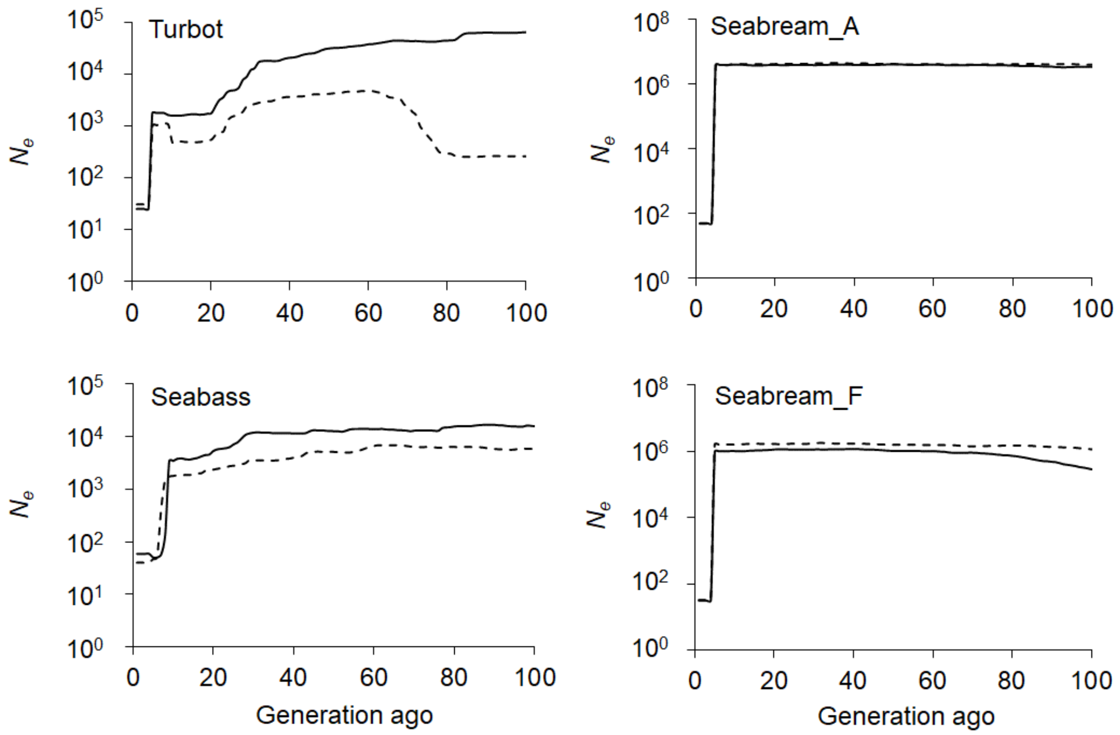

Supplement: Supplementary file 1 — Additional file 1. Estimates of Ne (logarithmic scale) across the last 100 generations for each population analysed. Straight lines represent estimates obtained using data from parents and dashed lines represent estimates obtained using data from offspring. [file 12711_2021_680_MOESM1_ESM.pdf]
