# Supplementary material for: Estimates of recent and historical effective population size in turbot, seabream, seabass and carp selective breeding programmes
Source: Genet Sel Evol. 2021 Nov 6;53:85. doi: 10.1186/s12711-021-00680-9 (PMC8572424; doi:10.1186/s12711-021-00680-9)

Estimates of N_e (logarithmic scale) obtained with the LD method of Hayes et al. [5] across the last 100 generations for each population analysed.

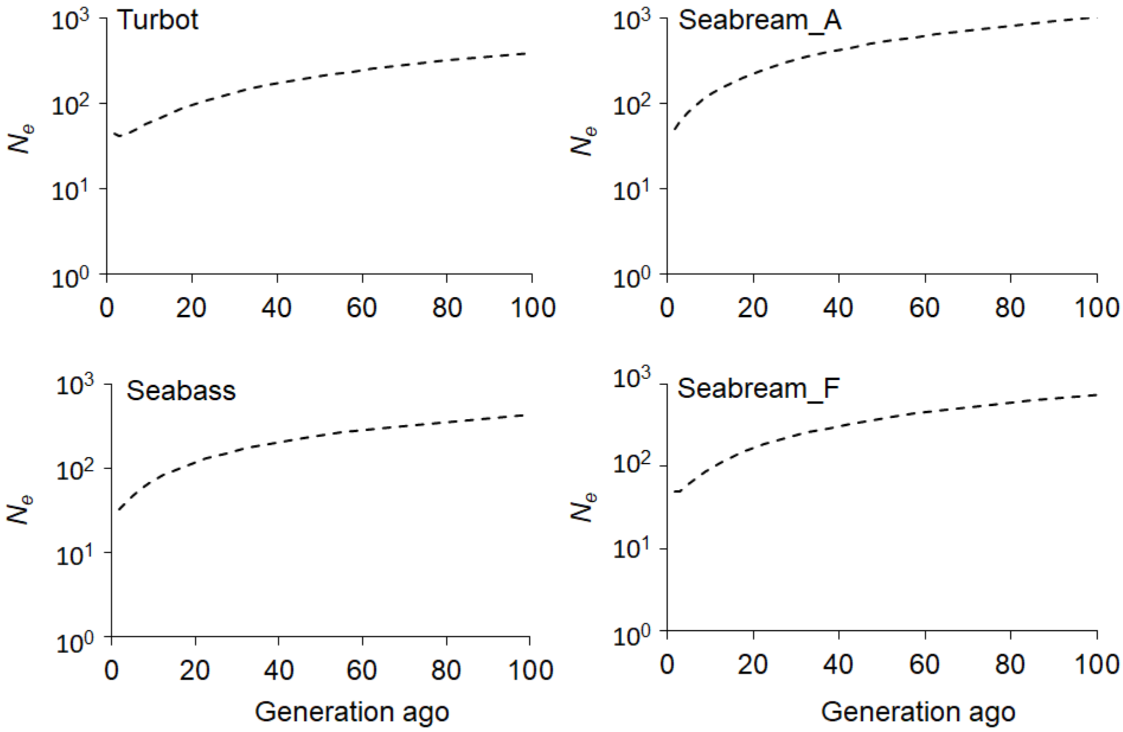

Supplement: Supplementary file 2 — Additional file 2. Estimates of Ne (logarithmic scale) obtained with the LD method of Hayes et al. [5] across the last 100 generations for each population analysed. [file 12711_2021_680_MOESM2_ESM.pdf]
