# Supplementary material for: Estimates of recent and historical effective population size in turbot, seabream, seabass and carp selective breeding programmes
Source: Genet Sel Evol. 2021 Nov 6;53:85. doi: 10.1186/s12711-021-00680-9 (PMC8572424; doi:10.1186/s12711-021-00680-9)

Estimates of historical effective population size from computer simulations

We used the software SLiM3 to simulate a genome nucleotide sequence of 200 Mb for a population of a sexually reproducing diploid species with random mating and non-overlapping generations with population size N = 1000 or 10,000 run for 10,000 generations. Neutral mutations were assumed to occur with rate 0.5×10^{-8} per nucleotide and generation, with a recombination frequency between consecutive nucleotides of 2.5×10^{-8}, thus implying an average rate of recombination of 2.5 cM / Mb, analogous to that found in the fish species analysed in the main text. Ten and five generations before the last one (for the scenarios of N = 1000 and N = 10,000, respectively) a sudden drop in population size down to N = 100 and N = 50 individuals occurred, and this population size was maintained for the last nine and four generations, respectively. In the last generation, the genotypes of 20,000 SNPs in the 100 individuals were analysed for linkage disequilibrium to obtain historical estimates of the effective size with the programs GONE and the previous method from Hayes et al. [5]. Twenty simulated replicates were carried out.

The results are shown in Figure S3. The estimated historical N_e obtained with the program GONE was able to infer the drastic drops to N = 100 (Figure S3a) and N = 50 (Figure S3b) individuals in the last ten and five generations (respectively), although with some variation around this value. With respect to the estimation of the ancestral N_e (before the sudden drop), the estimates were unbiased with a certain variation around the true value in the case with an ancestral population of size N = 1000 individuals. However, estimates of the ancestral N_e in the case of N = 10,000 showed a tendency to be upwardly biased, with values in some replicates up to around 50,000 individuals.

Estimates obtained by the method of Hayes et al. [5] were unable to detect the drastic drop in N_e, and showed a linear decline (curvilinear because of the logarithmic scale). In the earliest generation showed in the figure (generation 100 in the past), the estimates of the ancestral N_e were much lower than the true ones.

Figure S3. Estimates of N_e (logarithmic scale) obtained by computer simulations with the LD methods of of Hayes et al. [5] and Santiago et al. [6] for the scenarios of 1000 (a) and 10,000 (b) individuals simulated, respectively. Red solid line: real data; black solid lines: Hayes et al. [5]; black dotted lines: Santiago et al. [6].

(a)

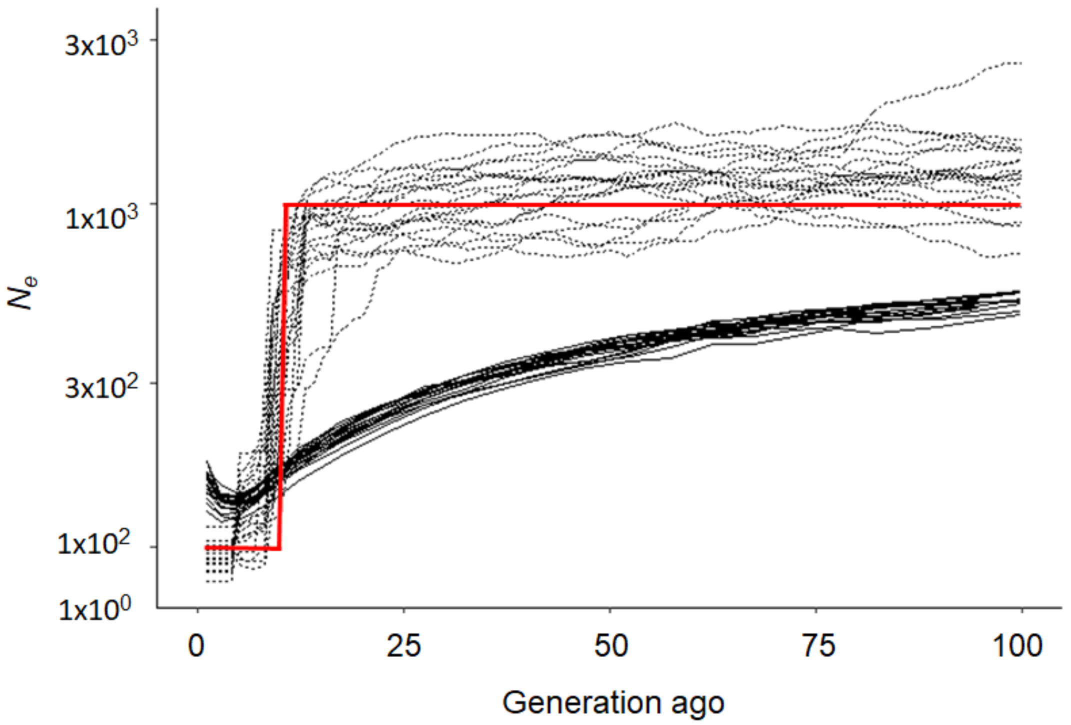

(b)

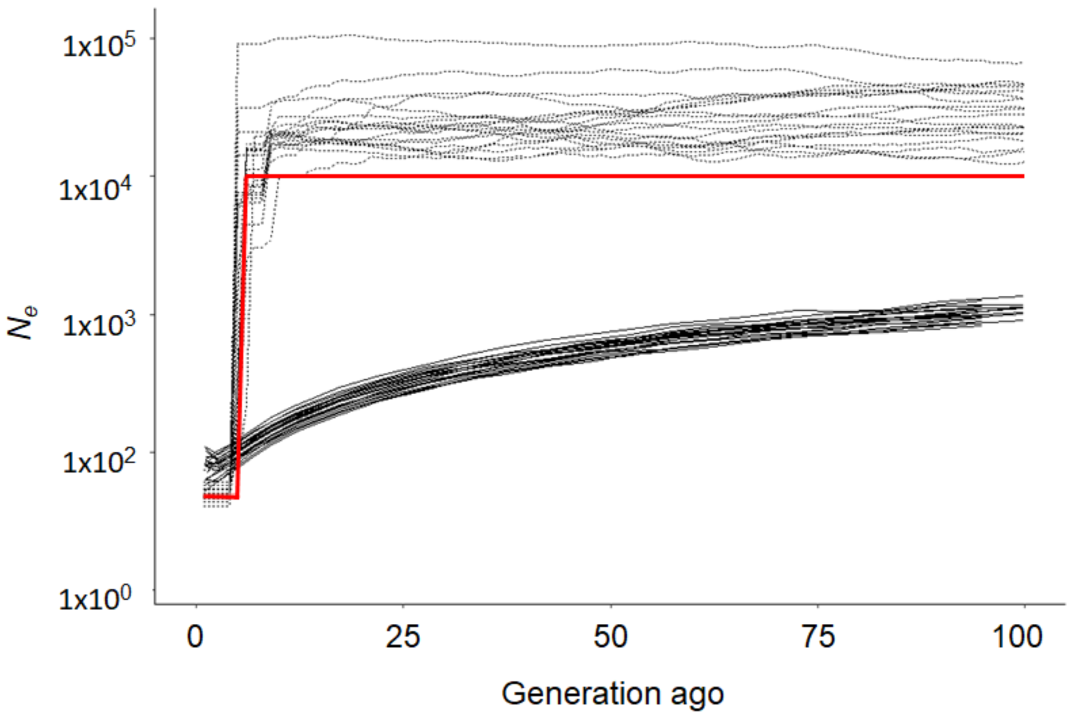

Supplement: Supplementary file 3 — Additional file 3. Estimates of Ne (logarithmic scale) obtained by computer simulations with the LD methods of Hayes et al. [5] and Santiago et al. [6], using the software SLiM3 [48]. [file 12711_2021_680_MOESM3_ESM.pdf]
